# Pancreatic imaging using an antibody fragment targeting the zinc transporter type 8: a direct comparison with radio-iodinated Exendin-4

**DOI:** 10.1007/s00592-017-1059-x

**Published:** 2017-10-24

**Authors:** Olof Eriksson, Olle Korsgren, Ram Kumar Selvaraju, Marjorie Mollaret, Yann de Boysson, Fabrice Chimienti, Mohamed Altai

**Affiliations:** 1 Department of Medicinal Chemistry, Uppsala University, 751 83 Uppsala, Sweden; 2 Rudbeck Laboratory, Department of Immunology, Genetics and Pathology, Uppsala University, 75185 Uppsala, Sweden; 3 Mellitech SAS, 38028 Grenoble, France; 4 Present Address: Innovative Medicines and Early Development Biotech Unit (IMED Biotech), AstraZeneca AB, 431 50 Mölndal, Sweden

**Keywords:** Beta cell imaging, Zinc Transport type 8, Type 2 diabetes, Imaging, Ab31, Islet imaging

## Abstract

**Aim:**

The zinc transporter 8 (ZnT8) has been suggested as a suitable target for non-invasive visualization of the functional pancreatic beta cell mass, due to both its pancreatic beta cell restricted expression and tight involvement in insulin secretion.

**Methods:**

In order to examine the potential of ZnT8 as a surrogate target for beta cell mass, we performed mRNA transcription analysis in pancreatic compartments. A novel ZnT8 targeting antibody fragment Ab31 was radiolabeled with iodine-125, and evaluated by in vitro autoradiography in insulinoma and pancreas as well as by in vivo biodistribution. The evaluation was performed in a direct comparison with radio-iodinated Exendin-4.

**Results:**

Transcription of the ZnT8 mRNA was higher in islets of Langerhans compared to exocrine tissue. Ab31 targeted ZnT8 in the cytosol and on the plasma membrane with 108 nM affinity. Ab31 was successfully radiolabeled with iodine-125 with high yield and > 95% purity. [^125^I]Ab31 binding to insulinoma and pancreas was higher than for [^125^I]Exendin-4, but could only by partially competed away by 200 nM Ab31 in excess. The in vivo uptake of [^125^I]Ab31 was higher than [^125^I]Exendin-4 in most tissues, mainly due to slower clearance from blood.

**Conclusions:**

We report a first-in-class ZnT8 imaging ligand for pancreatic imaging. Development with respect to ligand miniaturization and radionuclide selection is required for further progress. Transcription analysis indicates ZnT8 as a suitable target for visualization of the human endocrine pancreas.

## Introduction

The zinc transporter 8 (ZnT8, product of the solute carrier family 30 member 8 (SLC30A8) gene) has been suggested as a suitable target for non-invasive visualization of the functional pancreatic beta cell mass [1, 2].

ZnT8 is a member of the ten zinc transporters family that catalyzes the extrusion of Zn^2+^ from the cell cytosol into the extracellular space or intracellular organelles. Insulin is stored within secretory vesicles, crystallized as zinc-insulin hexamers. ZnT8 is thus mostly expressed on the insulin granules, as well as on the plasma membrane [3]. It is highly evolutionary conserved with a 98% amino acid homology in rodents and non-human primates to human ZnT8 [4].

ZnT8 is required by the beta cell for correctly storing of insulin molecules within the intracellular vesicles [5]. ZnT8 deficiency leads to diabetes like pathology in mice on high-fat diet, showing the importance of ZnT8. ZnT8 deficiency in mice fed standard diet, however, did not affect body weight, fasting b-glucose nor insulin sensitivity, but impaired the response to glucose tolerance test [6]. The presence of ZnT8 autoantibodies has been shown to predict development of T1D [7], and genetic variations in the SLC30A8 locus have been linked to susceptibility to type 2 diabetes [8, 9]. Presence of ZnT8 therefore seems tightly connected to several processes involved in the normal physiology of the beta cell.

Reports reveal that ZnT8 expression is restricted to the islets of Langerhans, and in particular the beta cells [3, 5, 10]. On this basis, ZnT8 has thus been suggested as a theoretically interesting target for human beta cell imaging. The main obstacle for pursuing this avenue has been the lack of available ligands suitable for radiolabeling. Mono- and polyclonal antibodies targeting ZnT8 epitopes will have slow biodistribution and necessitate labeling with radionuclide with several days of half-life where the associated radiation dose to healthy individuals and individuals with diabetes would be unacceptable.

Recently, several small antibodies targeting one of the ZnT8 loops that become accessible to the beta cell membrane during insulin-secreting events have been generated. Antibody screening was performed on non-permeabilized cells, suggesting that the generated antibodies can reach the ZnT8 protein at the cell surface. Antibody evaluation and miniaturization identified a lead compound Ab31, an antibody F(ab) targeting the loop 2 (ACERLLYPDYQIQATV) of human ZnT8, suitable for imaging studies.

Here, we present a first-in-class ZnT8 radioligand for pancreatic imaging, evaluated in a direct comparison with radio-iodinated state-of-the-art Exendin-4.

## Materials and methods

### Transcription of SLC30A8 in pancreatic compartments

RNA preparation and analysis were conducted within the Human Protein Atlas project [http://www.proteinatlas.org/]. Isolated human islet and exocrine preparations from five donor pancreases stored in −70 °C, as well as fresh frozen pancreatic tissue embedded in OCT compound (Sakura Finetek, Alphen aan den Rijn, The Netherlands) was used as source of RNA. The use of human tissues was approved by the Uppsala Ethical Review Board (#2011/473, #Ups 02-577), and tissues were obtained from Uppsala Biobank.

RNA was extracted using the RNeasy mini kit (Qiagen) according to the manufacturer’s instructions. Disruption was conducted using a 3-mm steel grinding ball (VWR, Radnor, PA) and vortexing. Concentration and RNA integrity (RIN) were determined by Qubit 2.0 Fluorometer (Life Technologies) and Agilent 2100 Bioanalyzer (Agilent Technologies, Santa Clara, CA), respectively. Purity of the samples was confirmed by an A260/A280 value over 2.0 using NanoDrop (Thermo Scientific, Wilmington, DE). Samples with RIN values above 7.5 were sequenced by Illumina HiSeq 2000 and 2500 (Illumina, San Diego, CA) using the standard Illumina RNA-Seq protocol with a read length of 2 × 100 bases.

The raw reads obtained from the sequencing system were trimmed for low quality ends with the software sickle [11], using a Phred quality threshold of 20. All reads shorter than 54 bp after the trimming were discarded. The processed reads were mapped to the GRCh37 version of the human genome with Tophat v2.0.3 [12]. Potential PCR duplicates were eliminated using the MarkDuplicates module of Picard 1.77 [13]. To obtain quantification scores for all human genes, FPKM (fragments per kilobase of exon model per million mapped reads) values were calculated using Cufflinks v2.0.2 [14], which corrects for transcript length and the total number of mapped reads from the library to compensate for different read depths for different samples. The gene models from Ensembl build 69 [15] were used in Cufflinks. In addition to Cufflinks, HTSeq v0.5.1 was run to calculate read counts for each gene, which were used for analyses of differentially expressed genes using the DESeq package [16]. All data were analyzed using R Statistical Environment [17] with the addition of package ‘gplots’ [18]. For analyses performed in this study where a log2 scale of the data was used, pseudo-counts of + 1 were added to the data set.

### Ab31 affinity determination using ELISA

Ab31, a human F(ab) antibody targeting the loop 2 of human ZnT8 (46 kDa), was provided by Mellitech (Grenoble, France).

A 384-well Nunc^®^Maxisorp™ MTP, black, flat bottom, PS (Thermo Scientific, 10395991) was coated with 20 μL/well of antigen (hZnT8-2L-Trf proteins, loop 2 of ZnT8 fused to Trf) at 5 μg/mL in PBS and incubated overnight at 4 °C. The wells were then washed twice with PBST (PBS with 0.05% Tween^®^ 20). For blocking, 100 μL of 5% non-fat dry milk in PBST was added followed by incubation for 1–2 h at room temperature (RT). The wells were washed twice with PBST before the addition of the primary Ab (HuCAL-Fab). Twenty μL/well of Ab31 at concentration of 2 μg/mL (44 nmol/L) in PBST or HISPEC Assay Diluent (AbD Serotec, BUF049) was added followed by 1-h incubation at RT. The plates were then washed 5 times with PBST. Thereafter the secondary anti-Fab-AP conjugate (20 μL/well) (AbD Serotec, STAR126A) was added at 1:5000 dilution in HISPEC Assay Diluent. The wells were incubated for 1 h at RT before washing 5 times with PBST. For detection, a 20 μL/well AttoPhos^®^ (Roche, 1681982) was added at 1:10 dilution in H_2_O. The reader settings were excitations at 440 ± 25 nm and emission at 550 ± 35 nm. Values on unrelated antigens (e.g., BSA, CD33, GST) are used for the calculation of background.

### In vitro binding in MIN6 cells

MIN6 beta cells were seeded onto glass coverslips and allowed to attach for 72 h before fixation in 4% formaldehyde in PBS. After washing with 0.25% NH4Cl in PBS, the cells were permeabilized in PBS containing 0.1% Triton X-100. Cells were then blocked in 2% BSA in PBS for 1 h, followed by the addition of the Ab31 antibody fragment targeting ZnT8. Cells were washed in blocking buffer and further incubated with goat anti-human F(ab) fragment specific-Cy3 conjugate (Jackson ImmunoResearch), diluted in blocking buffer at 1:500. Cells were washed in PBS followed by a final wash in ddH_2_O. For immunolabeling without permeabilization, cells were incubated with Ab31 in PBS containing 0.1% bovine serum albumin for 30 min before fixation. Cells were then washed in PBS, and fixation and secondary antibody labeling were conducted as with permeabilized cells.

### Radiolabeling of an antibody fragment

#### Ab31 with iodine-125

Labeling of Ab31 with ^125^I (*t*_1/2_ = 59.4 days) was performed using direct iodination method [19]. For this 50 µg of Ab31 (1 µg in 1 µL PBS; 22 µmol/L) was incubated with 30 MBq Na^125^I. To start the reaction, 15 μl of Chloramine-T solution in PBS (4 mg/mL; 14 mmol/L) was added to the mixture of ^125^I and Ab31. The mixture was vortexed carefully and incubated for 60 s at RT. To stop the reaction, 15 μl of sodium metabisulfite solution in PBS (8 mg/mL; 41 mmol/L) was added to the reaction mixture and carefully vortexed. Radiochemical yield and purity were determined using silica-impregnated ITLC strips (150–771 DARK GREEN Tec-Control Chromatography strips, Biodex Medical Systems) eluted with 80% acetone. The mixture was purified using size exclusion NAP-5 columns (GE Healthcare Life Sciences, Uppsala, Sweden).

### Radiolabeling of Exendin-4 with iodine-125

The synthetic peptide agonist E7144-Exendin-4 (39a.a.), a potent GLP-1 agonist, was obtained from Sigma-Aldrich, St. Louis, Mo, USA. Exendin-4 was radio-iodinated directly via IODO-GEN method [19]. For that Exendin-4 15 µg was buffered in 120 µL 0.05 M tris buffer, pH 8.5. The Exendin-4 solution was transferred to IODO-GEN-coated tube (Pierce Biotechnology, IL, USA). In another tube, 30 MBq of 125I was mixed with 3.1 µL 0.01 M NaOH and the mixture was transferred to the IODO-GEN tube to start the reaction. The mixture was incubated for 60 min at RT with careful vortexing. The reaction is stopped by removing the reaction solution from the IODO-GEN tube. The radiochemical yield and purity were determined using ITLC strips as mentioned above.

### In vitro autoradiography

Sections (20 µm thick) were processed from frozen (−80 °C) explanted INS-1 tumors (immortalized rat beta cell line inoculated in immunodeficient nu/nu mice, *n* = 2 [20]), as well as from frozen pancreas from non-diabetic rat (*n* = 1, male, Sprague–Dawley). The procedures were performed in accordance with national guidelines and were approved by the local ethics committee for animal research.

Sections were also processed from frozen pancreatic biopsies from deceased human donors (non-diabetic individuals and individuals with T1D and T2D). The use of human tissues was approved by the Uppsala Ethical Review Board (#2015/401) and tissues obtained from Uppsala Biobank.

All sections were pre-incubated in 100 mL 50 mM PBS (pH 7.4) for 10 min. Then, radioactivity corresponding to 1–2 nM [^125^I]Ab31 or [^125^I]Exendin-4 was added. The sections were incubated for 120 min at RT. Non-displaceable binding of [^125^I]Ab31 was assessed in a separate assay, by co-incubation with 200 nM unlabeled Ab31. Non-displaceable binding of [^125^I] Exendin-4 was assessed in yet another separate assay, by co-incubation with 200 nM unlabeled Exendin-4 peptide.

After incubation, tissue sections were washed 3 times for 4 min in 150 mL 50 mM PBS at 4 °C before being dried at 37 °C for 15 min. The sections were exposed against a phosphorimager screen for 120 min, together with a 10 µL of known reference radioactivity cross-calibrated against a gamma-counter. The plates were digitalized using a Cyclone Plus Phosphorimager (PerkinElmer) at 600 dpi and analyzed using ImageJ (NIH).

Regions of interest (ROIs) were drawn over the entire INS-1 or pancreas sections, and ROI uptake first calibrated to Bq/mm^2^ by the reference and then to Bq/mm^3^ by the specific radioactivity and section thickness. Specific binding was defined by subtracting non-displaceable binding from total binding.

### In vivo biodistribution

Sprague–Dawley rats (*n* = 10, 472 ± 22 g) were kept under standard laboratory condition with unlimited access to chow and water. The animal experiments were approved by the Local Ethics Committee for Animal Research and were performed in accordance with local institutional and Swedish national guidelines and regulations.

Sedated animals (isoflurane 2.5–3.0%) were administered 25 kBq (92 µg) [^125^I]Ab31 per rat (*n* = 5, corresponding to 2 nmol antibody fragment per rat) or 25 kBq (0.04 µg) [^125^I]Exendin4 per rat (*n* = 4, corresponding to 0.001 nmol peptide) intravenously through the tail vein. The animals were allowed to wake up after tracer administration and were sacrificed after either 1 h (*n* = 3 for [^125^I]Ab31, *n* = 4 for [^125^I]Exendin4) or 5 h (*n* = 3 [^125^I]Ab31 only) by CO_2_ (70%). Tissues were excised postmortem, weighed and measured for radioactivity in an automated gamma-counter with 3-inch NaI(Tl) detector (PerkinElmer). The tissue uptake was corrected for tissue weight and total administered tracer dose and expressed as percentage of injected activity being taken up in each tissue per gram (%ID/g).

### Statistical considerations

Data on group level are reported as mean ± SEM. Statistical analysis was performed in GraphPad Prism 5 (GraphPad, La Jolla, CA, USA). Differences between multiple groups were assessed by the one-way ANOVA with Tukey’s multiple comparisons test using a significance level of *p* < 0.05.

## Results

### Transcription of SLC30A8 in pancreatic compartments

**Fig. 1:**
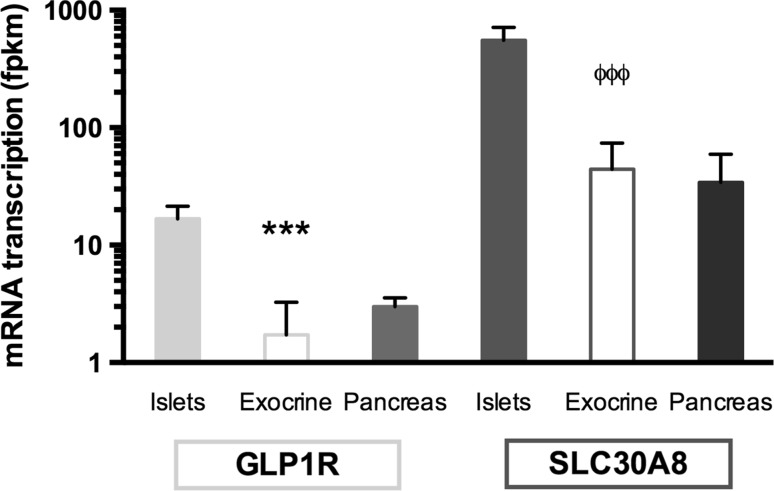
mRNA transcription of SLC30A8 in different pancreatic compartments, comparison with GLP1R. * indicates difference in comparison with the Islet transcription of GLP1R. ϕ indicates difference in comparison with the islet transcription of SLC30A8. Note that the y-axis is logarithmic

RNA analysis of the pancreas compartments showed higher transcript levels of SLC30A8 in islets (554.2 FPKM) compared to exocrine tissue (44.3 FPKM) (*p* < 0.001) (Fig. 1). Similarly, GLP1R transcription was higher in islets (16.7 FPKM) than in exocrine tissue (1.7 FPKM) (*p* < 0.001). The transcription of SLC30A8 was a magnitude greater than GLP1R in islets.

### Affinity of Ab31 to antigen and in vitro binding in MIN6 cells

The affinity for Ab31 for the loop 2 of the ZnT8 was 108 nM as determined by competition ELISA.

**Fig. 2:**
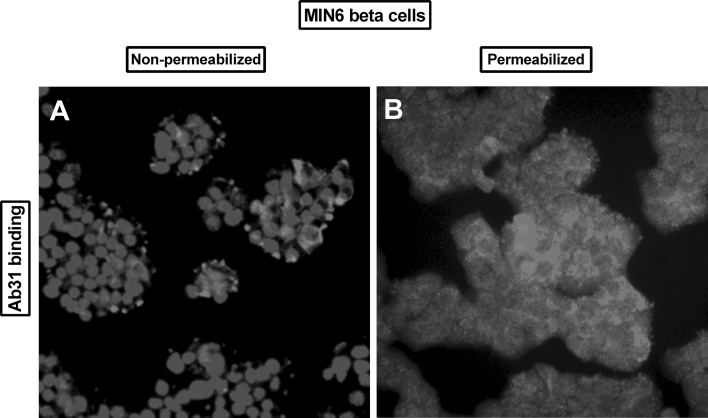
Fluorescent microscopy of in vitro binding of Ab31 to non-permeabilized (**a**) and non-permeabilized **b** MIN6 beta cells. Pseudo-color overlay images of Ab31 binding (red) and cell nucleus (DAPI, blue). Cells were imaged at 320× magnification under an inverted fluorescence microscope (Zeiss)

Ab31 binding to non-permeabilized MIN6 cells was concentrated to the plasma membrane on the cell surface (Fig. 2). Permeabilization resulted in strong binding also within the cytosol on secretory vesicles.

### Radiolabeling of Ab31 and Exendin-4 with iodine-125

The labeling yield was 75 ± 9% for [^125^I]Ab31 and 57 ± 2 for [^125^I]Exendin-4. Purification using NAP-5 size exclusion columns provided a radiochemical purity of more than 95% for both labeled molecules.

### In vitro autoradiography

**Fig. 3:**
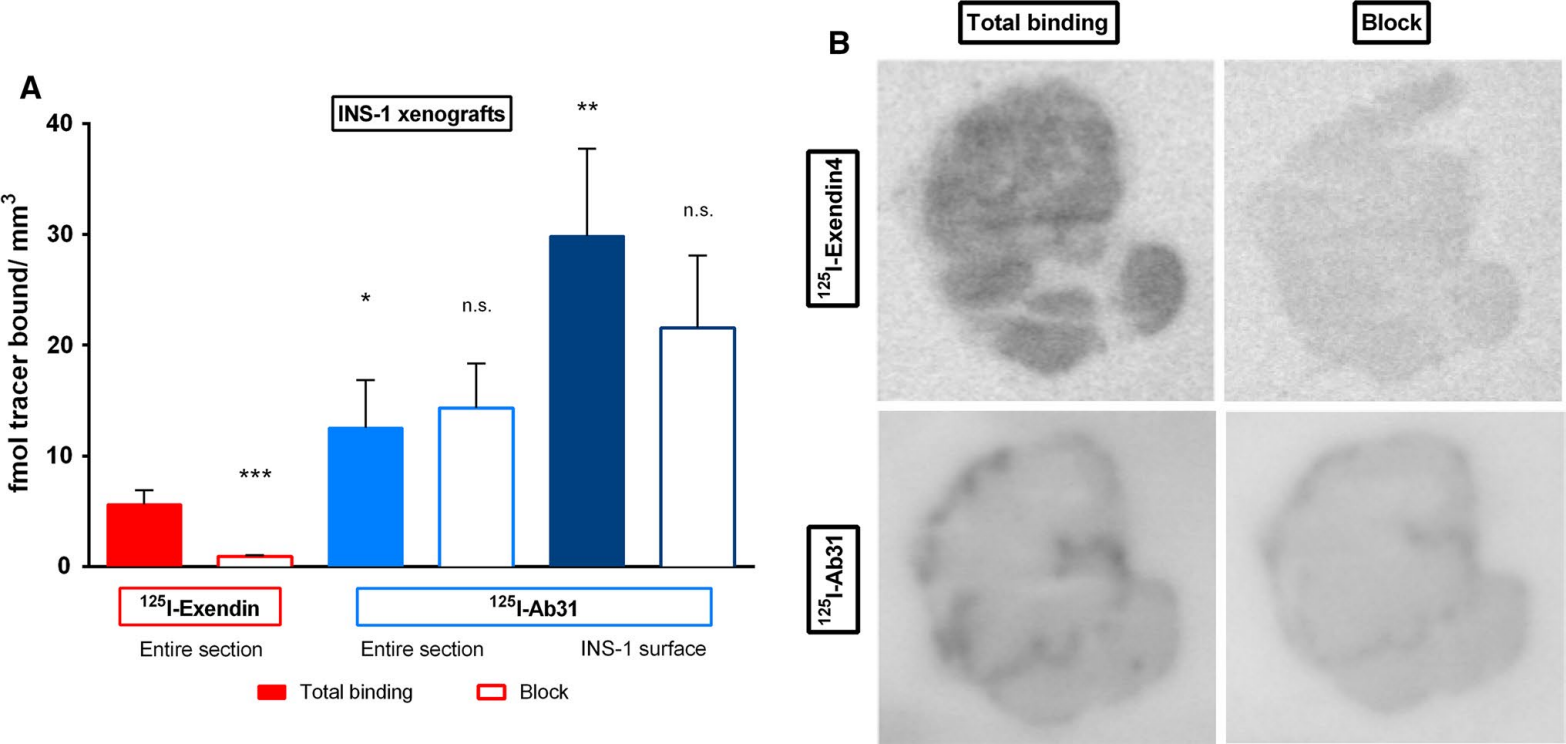
In vitro autoradiography of nanomolar concentrations [^125^I]Ab31 and [^125^I]Exendin-4 on INS-1 (beta cell origin) xenograft sections. **a** Total and non-specific binding of [^125^I]Ab31 and [^125^I]Exendin-4 expressed as fmol bound per area, either in the entire tissue section, or in the case of [^125^I]Ab31 also on the tumor surface. **b** Representative autoradiograms of the tissue sections. Asterisk indicates difference in binding in comparison with the total binding of [^125^I]Exendin-4

Total binding of [^125^I]Ab31 in fmol/mm^2^ on INS-1 sections was higher compared to [^125^I]Exendin-4 (*p* < 0.05) (Fig. 3a). Binding of [^125^I]Ab31 was heterogeneous in the tumor, appearing concentrated on the surface of the INS-1 sections (Fig. 3b). Binding of [^125^I]Ab31 in the surface regions was almost 6 times higher than for [^125^I]Exendin-4 (*p* < 0.01). Co-incubation with 200 nM unlabeled Ab31 had a tendency to decrease binding of [^125^I]Ab31 in the high uptake surface areas.

The binding of [^125^I]Exendin-4 was homogenous across INS-1 sections and displaceable by 200 nM unlabeled Exendin-4 (*p* < 0.001).

**Fig. 4:**
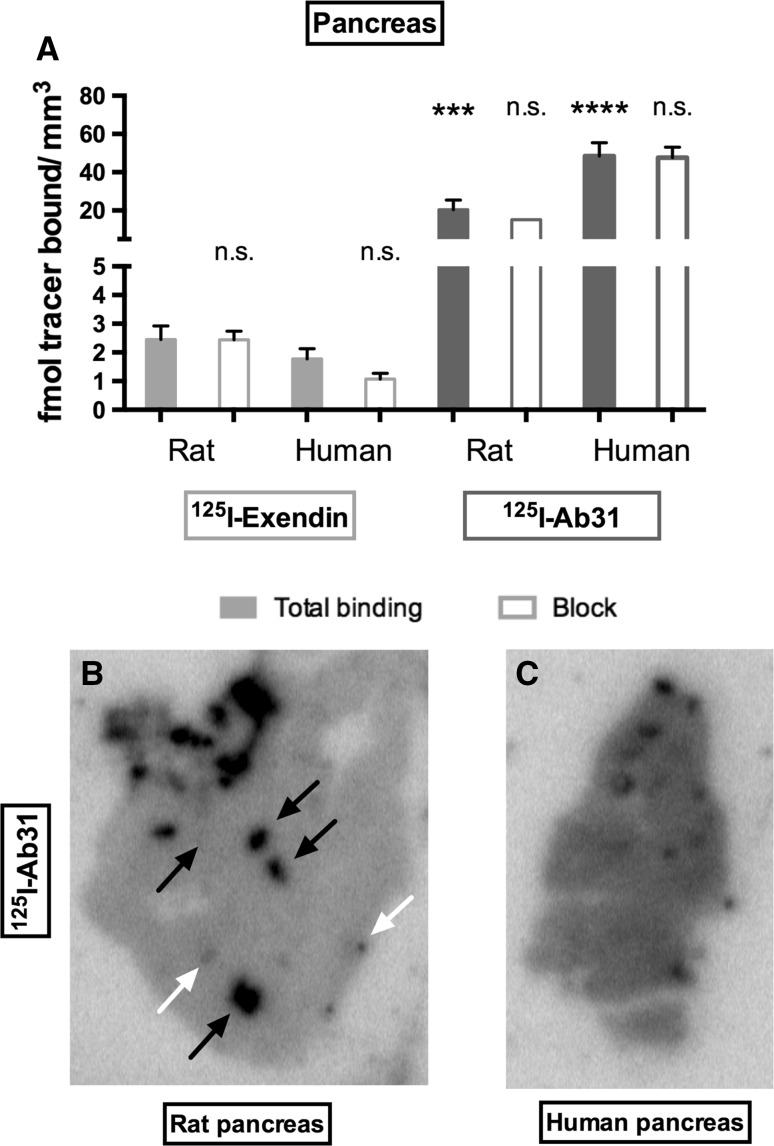
In vitro autoradiography of [^125^I]Ab31 and [^125^I]Exendin-4 on pancreas sections from non-diabetic rat and human. **a** Quantified binding in fmol/mm^2^ both in absence and presence of 200 nM of the unlabeled ligand. Asterisk indicates difference in binding in comparison with the total binding of [^125^I]Exendin-4 in each species. Representative autoradiograms of [^125^I]Ab31 binding on sections from rat pancreas (**b**) and human pancreas (**c**)

Similarly, total binding of [^125^I]Ab31 on rat (*p* < 0.001) and human (*p* < 0.0001) pancreas was greater than for [^125^I]Exendin-4, consistent with the transcription levels of the respective gene in human pancreas (Fig. 4a). The binding pattern of [^125^I]Ab31 on pancreatic sections was highly heterogeneous (Fig. 4b, c). Comparison with adjacent hematoxylin (HTX)-stained rat sections showed that the strongest focal uptake represented ducts (black arrows, Fig. 4b), while islets of Langerhans sometimes exhibited noticeably elevated binding above the background, but low in comparison with the ducts (white arrows, Fig. 4b).

### In vivo biodistribution

**Fig. 5:**
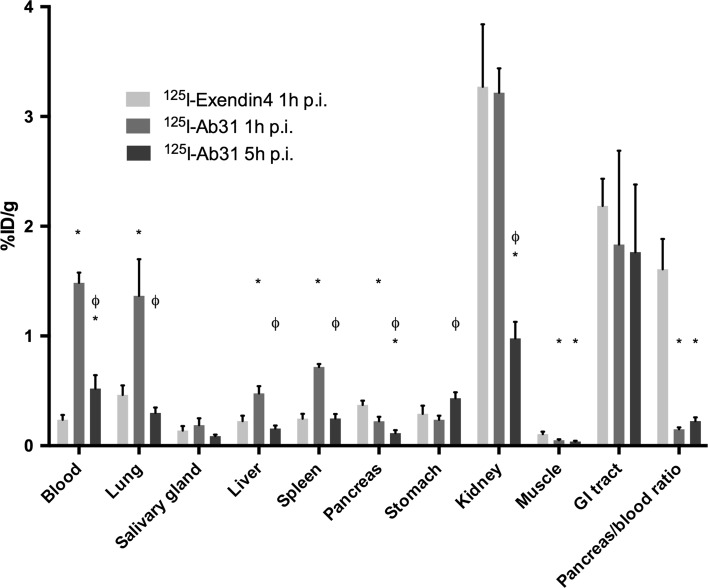
In vivo biodistribution of [^125^I]Ab31 in a direct comparison with [^125^I]Exendin-4. * indicates difference in uptake in comparison with [^125^I]Exendin-4. ϕ indicates difference in [^125^I]Ab31 uptake at the 5 h p.i. time point in comparison with 1 h p.i

Results from the comparative in vivo biodistribution study of [^125^I]Ab31 and [^125^I]Exendin-4 in Sprague–Dawley rats are summarized in Fig. 5. The data showed low radioactivity concentration of [^125^I]Exendin-4 in the blood (0.24 ± 0.04%ID/g) and most body organs except for the kidney and GI tract. The uptake in the pancreas was 0.37 ± 0.04%ID/g, and the pancreas-to-blood ratio was 1.61 ± 0.27. The concentration of radioactivity in the blood 1 h after the injection of [^125^I]Ab31 was 1.49 ± 0.09%ID/g but decreased to 0.52 ± 0.12%ID/g after 5 h. The elimination half-life in blood was 1.8 h. Higher compound levels of [^125^I]Ab31 compared to [^125^I]Exendin-4 were seen in all collected tissues. The kidney uptake was the highest among all studied body organs at this time point 3.21 ± 0.22%ID/g and was not significantly different from [^125^I]Exendin-4 (3.27 ± 0.56%ID/g). [^125^I]Ab31 cleared from most studied organs 5 h p.i. The uptake of [^125^I]Ab31 in the pancreas was 0.23 ± 0.04%ID/g 1 h p.i., and the pancreas-to-blood ratio was 0.15 ± 0.02. The uptake of [^125^I]Ab31 in the pancreas decreased by ca. twofold (0.115 ± 0.02%ID/g) 5 h p.i. However, the pancreas-to-blood ratio increased slightly (*p* < 0.05) at this time point (0.23 ± 0.035).

## Discussion

Here we demonstrate that labeling of an anti-ZnT8 F(ab) fragment Ab31 was possible using iodine-125. Labeling permitted the characterization of the targeting agent both in vitro and in vivo. For comparative purposes, Exendin-4 was also labeled with ^125^I, as GLP-1R targeting is considered one of the most promising approaches for human beta cell imaging [21].

SLC30A8 mRNA was strongly and consistently transcribed in human islets of Langerhans, consistent with earlier reports of beta cell restriction of this transporter. The islet specific transcription was magnitudes higher than for GLP-1R, providing further evidence of ZnT8 as a promising imaging target. The exocrine transcription pattern, although lower than in islets, was high in comparison with GLP-1R and thus high pancreas background ZnT8-mediated binding may pose an issue for beta cell visualization.

The affinity of Ab31 as determined by competition ELISA was 108 nM. This is in line with other monovalent antibodies used in other indications, for example, oncology. There, successful imaging of tumors has been achieved using antibody fragments with affinities ranging from 9 to 300 nM, respectively [22, 23]. Previous work has also shown that quantitative evaluation of pancreatic beta cell mass was possible using K14D10 IgG and its Fab fragment, both of which have an affinity of 6–20 nM [24].

[^125^I]Ab31 exhibited strong binding to INS-1 sections compared to [^125^I]Exendin-4 (*p* < 0.05). However, co-incubation with 200 nM Ab31 did not reduce the binding considerably, either in the concentrated binding on the INS-1 xenograft surface or in the tumor core. Given the approximately 100 nM affinity of Ab31, it is possible that higher excess of unlabeled Ab31 is required for a substantial displacement effect.

Binding of [^125^I]Ab31 on pancreatic sections was a magnitude higher than [^125^I]Exendin-4 as predicted from the mRNA transcription results (*p* < 0.001). Again, co-incubation with 200 nM Ab31 did not produce a blocking effect on the binding. Importantly, focal binding patterns (the approximate size of islets of Langerhans) were seen in both rat and human pancreas, which was not seen on INS-1 sections. However, islets of Langerhans as identified by HTX staining did only account for some of the focal uptake patterns, the remaining representing pancreatic ducts. While ZnT8 expression in ducts has not previously been reported, zinc signaling (through GPR39) has been reported as part of the pancreatic duct biology [25]. Rodent adult duct lining can in theory reprogram into insulin-secreting β-like cells, possibly including expression of ZnT8 [26]. However, it seems unlikely that such rare phenomenon should account for the strong duct uptake seen here. The expression of ZnT8 and possible role in pancreatic ducts remains to be confirmed.

[^125^I]Exendin-4 binding to INS-1 sections could be competed away by unlabeled Exendin-4 in excess, confirming specificity to the GLP-1R. On the other hand, binding of [^125^I]Exendin-4 to pancreas sections from rat and human was only partially competed away in the in vitro autoradiography assay. This is presumably due to issues inherent to working with the pancreas in particular: Warm ischemia time (especially important in human donor pancreases) may affect GLP-1R function as well as the possible presence of proteases from exocrine pancreas. A combination of these factors likely contributes to the limited in vitro blocking effect seen here in rat and human pancreas.

The biodistribution profile of [^125^I]Ab31 (46 kDa) showed an initial elevated uptake in blood 1 h p.i (Fig. 5). which decreased by 5 h p.i. [^125^I]Ab31 concentration in the blood was higher than Exendin-4 (4 kDa). This can be attributed to the difference in clearance rate between the two molecules. A finding from the biodistribution of Ab31 is the high uptake in the lungs and spleen at 1 h p.i (1.36 ± 0.33 and 0.72 ± 0.03%ID/g, respectively). Transcription (but not protein expression) of the SLC30A8 gene in the lung, but not spleen, has been reported [27]. It would be reasonable to assume that the elevated uptake in the lungs may consist of a combination of ZnT8 receptor specific and non-specific interactions as well as vascular contribution from perfusion by [^125^I]Ab31-rich blood. The uptake in spleen is likely non-target-mediated and due to non-specific binding or high perfusion. This elevated uptake decreased significantly by 5 h in a manner similar to that of the blood (0.3 ± 0.05 and 0.09 ± 0.013%ID/g for lungs and spleen, respectively).

The kidney was the organ that had highest uptake of radioactivity. ZnT8 expression has been reported in kidney [28], but the high radioactivity signal also indicates renal excretion of both [^125^I]Ab31 and [^125^I]Exendin-4. This is expected for proteins smaller than the glomerular pore size (< 60 kDa). Compared to radiometal-labeled F(ab) or Exendin-4, the renal retention of our iodinated tracers was relatively low [29, 30]. This can be explained by the non-residualizing properties of the iodine label in the tubular cells. Although beyond the scope of this paper, iodination of Exendin-4 (or its analogues) may offer an alternative approach to decrease the high kidney retention of radiometal-labeled derivatives such as observed for Fluorine-18 [31]. This will consequently decrease unnecessary high radiation dose to the kidney and allow for more sensitive pre- and intraoperative detection of beta cell mass or insulinomas.

The in vivo uptake of [^125^I]Ab31 in the pancreas was low both after 1 and 5 p.i. However, this is not entirely unexpected if the target antigen is expressed only in the pancreatic beta cells which comprise 2–3% of the organ volume. Additionally, the beta cell specific retention in pancreas may be increased by a residualizing radionuclide such as Indium-111 or Gallium-68.

In vitro, Ab31 (*K*_D_ = 108 nM) binds permeabilized pancreatic beta MIN6 cells as confirmed by fluorescence microscopy (Fig. 2). However, we have not seen similar specific uptake of Ab31 in non-permeabilized INS-1 xenograft, human and rat pancreas sections. This was in agreement with the results from the in vivo biodistribution (Fig. 5). This difference may indicate that although both *K*_D_ of Ab31 and cellular specificity seemed suitable for in vivo use, other parameters, e.g., pancreatic uptake and retention of the tracer, binding sites and injected dose might be of importance and should be evaluated in follow-up studies.

Exendin-4, on the other hand, showed specific uptake in both INS-1 xenografts and human pancreas sections in vitro. This binding was significant. Results from the comparative biodistribution study demonstrated approximately twofold higher uptake of [^125^I]Exendin-4 in the pancreas compared to [^125^I]Ab31 (0.37 ± 0.02 vs. 0.22 ± 0.03%IA/g 1 h p.i.). The pancreas-to-blood ratio (a measure of imaging contrast) of [^125^I]Exendin-4 was ca. 12 times higher compared to that of [^125^I]Ab31 (1.7 ± 0.25 vs. 0.15 ± 0.018, respectively, at 1 h p.i.).

A surprising finding is the lower pancreas uptake of [^125^I]Exendin-4 in our study in comparison with another ^125^I-labeled Exendin-4 reported by Läppchen et al. [32]. This group have successfully labeled Exendin-4 with ^125^I, and the uptake of the tracer, [Nle^14^,^125^I-Tyr^40^-NH_2_]Ex-4, in the pancreas was 25.3 ± 4.2%IA/g at the same 1-h time point [32]. It is important to mention that Exendin-4 was labeled using two different methods in these studies. iodine-125 in the current study was bound to Exendin-4 through histidine residue on the N-terminal (His^1^) of the molecule, while a tyrosine residue was inserted in the C-terminus (Tyr^40^)—to permit iodination—in the second study. It was reported earlier that modification on the N-terminal end of Exendin-4 strongly affects the properties of the peptide [33]. Binding to GLP-1R was not impaired, but the peptide internalization was reduced by (20–33%) in contrast to other modified Exendin-4 derivatives. As a consequence, the uptake in GLP-1R positive organs was strongly reduced. This could perhaps explain the lower accumulation of our [^125^I]Exendin-4 in GLP-1R positive organs in comparison with [Nle^14^,^125^I-Tyr^40^-NH_2_]Ex-4, as well as the difficulty in measuring specific pancreatic binding in rat sections. Brom et al. [34] have also shown that Exendin-4, modified on the C-terminus (specifically Lys^40^), appeared to be less vulnerable to modifications. In that study, binding to GLP-1R positive organs was preserved using [Lys^40^(^111^In-DTPA)]Exendin-4. Moreover, the use of the residualizing ^111^In label compared to our ^125^I (non-residualizing) labeling may explain the higher radioactivity concentration in the pancreas observed in that study. Regardless of the underlying reason, it was obvious that [^125^I]Exendin-4 had superior in vitro and in vivo targeting properties to [^125^I]Ab31. A major issue with the use of ZnT8 as a target for beta cell imaging is that it is intracellularly located to a significant extent. Results from both our in vitro and in vivo characterization clearly indicate that detection of the target would be possible when it is displayed on the cell surface. This could be done in vitro by permeabilization but optimal in vivo targeting would be possible during insulin secretion, i.e., when ZnT8 loops become increasingly accessible to the beta cell membrane [35].

## Conclusion

We report a first-in-class ZnT8 imaging ligand for pancreatic imaging. An antibody derived imaging agent with appropriate size will offer repeated passages in serum and hence increase uptake and distribution of the tracer in targeted tissue while still maintaining an overall adequate imaging contrast. Results showed that [^125^I]Ab31 did not provide any improvement for beta cell imaging compared to [^125^I]Exendin-4. Development with respect to ligand miniaturization, affinity and radionuclide selection is required for further progress. Albeit current findings, transcription analysis indicates ZnT8 as a promising target for visualization of the human endocrine pancreas.
